# Analysis and Testing of Bisphenol A—Free Bio-Based Tannin Epoxy-Acrylic Adhesives

**DOI:** 10.3390/polym8040143

**Published:** 2016-04-15

**Authors:** Shayesteh Jahanshahi, Antonio Pizzi, Ali Abdulkhani, Alireza Shakeri

**Affiliations:** 1 Department of Wood and Paper Science and and Technology, University of Tehran, 31585-43414 Karaj, Iran; shjahanshahi@ut.ac.ir (S.J.); Abdolkhani@ut.ac.ir (A.A.); 2 Laboratoire d’Etude et de Recherche sur le Materiau Bois (LERMAB), University of Lorraine, Epinal 88000, France; 3 Department of Physics, King Abdulaziz University, Jeddah 21589, Saudi Arabia; 4 Department of Chemistry, University of Tehran, 6455-14155 Tehran, Iran; Alireza.Shakeri@ut.ac.ir

**Keywords:** tannin, biosourced adhesives, epoxy resin, glycidylation, acrylic acid, adhesives, MALDI-TOF, FT-MIR, ^13^C-NMR

## Abstract

A tannin-based epoxy acrylate resin was prepared from glycidyl ether tannin (GET) and acrylic acid. The influence of the reaction condition for producing tannin epoxy acrylate was studied by FT-MIR, ^13^C-NMR, MALDI-TOF spectroscopy and shear strength. The best reaction conditions for producing tannin epoxy acrylate resin without bisphenol A was by reaction between GET and acrylic acid in the presence of a catalyst and hydroquinone at 95 °C for 12 h. FT-MIR, ^13^C-NMR and MALDI-TOF analysis have confirmed that the resin has been prepared under these conditions. The joints bonded with this resin were tested for block shear strength. The results obtained indicated that the best strength performance was obtained by the bioepoxy-acrylate adhesive resin prepared at 95 °C for a 12-h reaction.

## 1. Introduction

Epoxy resins represent an important class of polymers primarily due to their versatility, high degree of crosslinking and ease of use. Thus, they are very widely used in modern industry, such as for adhesives, coatings and paints, substrates for printed circuit boards and encapsulating materials for microelectronic devices [1]. They contain many hydroxyl groups, and the nature of the interchain bonds gives cured epoxies many desirable characteristics [2]. These characteristics include excellent adhesion to many substrates, high strength, chemical and moisture resistance, low thermal shrinkage and high dielectric properties, fatigue resistance, corrosion resistance and electrical resistance [3]. Epoxy resins have the ability to transform their liquid (or thermoplastic) state to tough, hard thermoset solids. This hardening is accomplished by the addition of a chemically-active curing agent [4]. Epoxy resins were also produced by the reaction of the corresponding biobased polyols and petroleum-based epichlorohydrin [5,6,7,8]. Commercialized for more than 50 years, bisphenol A (BPA) is the most common phenol derivative used in epoxy resin formulations to produce adhesives, laminates, structural composites, protective coatings and many other products. BPA-based resins are also encountered in human health applications, such as filling materials or sealants in dentistry. However, these polymers are sensitive to hydrolysis and leaching of BPA, leading to widespread human exposure as revealed by a number of studies [9,10]. They are mostly derived from BPA, a compound classified as CMR (carcinogenic, mutagenic and reprotoxic). Moreover, high BPA levels in various human fluids and tissues have been detected, which may be responsible for health damages [11].

Recent awareness of BPA toxicity combined with the limitation and high cost of fossil resources implies necessary changes in the field of epoxy resins. Epoxy lignin has also been studied, the results showing that epoxy resin can be cured by lignin, and the curing temperature for the blends can be reduced by the introduction of a polyamine cure agent [12]. The reactivity of catechin toward epichlorohydrin to form glycidyl ether derivatives has also been studied. The glycidyl ether of catechin (GEC) thermal properties showed that these new synthesized epoxy resins displayed interesting properties compared to the commercial diglycidyl ether of bisphenol A (DGEBA) [5,6,7,8]. Major issues today are to find both alternatives to the typical synthesis route for epoxy resins and substitutes for BPA.

The growing interest in the utilization of renewable resources as an alternative to petroleum-based polymers has focused much attention on the development of polymeric materials from vegetables and renewable resources. Among the renewable, biosourced phenolic compounds being studied are condensed flavonoid tannins. Their source is from forestry or viticulture by-products, thus not in competition with food crops. Resins derived from functionalized natural compounds possess thermal and mechanical properties comparable to those of conventional fossil fuel-derived resins, such as the diglycidyl ether of BPA [5,6,7,8]. To cure epoxy resins, hardeners, such as an amine or an aldehyde, are usually needed. However, because in this work, a hardener is not used, it was preferred to concentrate on acrylate epoxy resins. The acrylate epoxies are a category of resins prepared with unsaturated carboxylic acids and in the presence of transesterification catalysts that renders the esterification reaction between the carboxyl group of the acid on the epoxide groups of the epoxy resin to form vinyl ester bonds possible.

Epoxy-acrylic resins have been particularly developed for self-stratifying coatings, thus coatings that do not need to be applied in successive layers. It is the presence of such a concentration gradient in the layer applied to a surface, thus the elimination of interlayers boundaries [13], that presents some interest also for the application of these materials as adhesives. This is so because elimination of the interlayers; boundary, which is a potential site of weakness and failure, may be of benefit to adhesives when these are applied in a single layer, single face. Furthermore, the lack of the need to use an epoxy hardener is of additional interest, as the epoxy acrylics are faster curing than epoxies alone, and this characteristic is of interest for adhesives, as well.

In this paper is described the synthesis of a novel bioepoxy resin based on a glycidyl ether tannin (GET) [6] reacted with acrylic acid, and the time and temperature reaction conditions of preparation are investigated, as well as the derived resin properties.

## 2. Material and Methods

### 2.1. Materials

**Figure 1:**
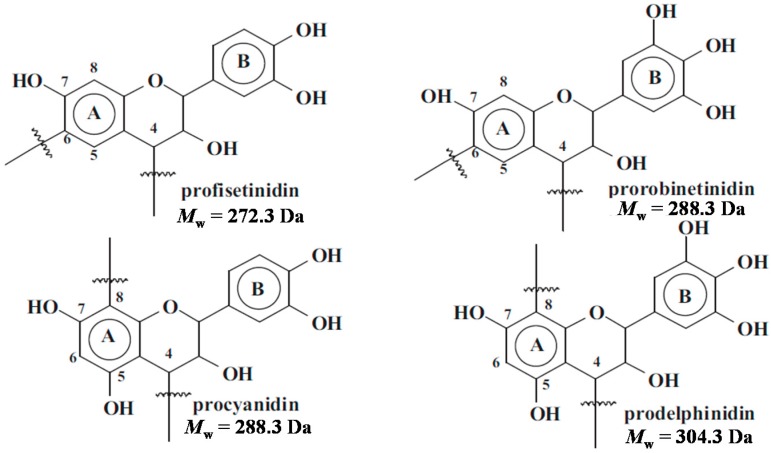
The four main structures in commercial flavonoid tannins.

Mimosa (Acacia mearnsii, formerly mollissima de Wildt) bark tannin extract was provided by Silva Chimica (San Michele Mondovì, Italy). It contained 80%–82% actual flavonoid monomers and oligomers, 1% of amino and imino acids, the balance being composed mainly of oligomeric carbohydrates, mainly hemicellulose fragments, and some carbohydrate monomers. The flavonoid part of mimosa tannin extract is composed in the majority of robinetinidin and fisetinidin, but also includes 10%–15% of catechin and delphinidin (Figure 1), each of these monomers forming the repeating units of the tannin, the units being linked, respectively, C4–C6 or C4–C8 [14]. The tannins’ enchainments formed by these units are called, respectively, prorobinetidin, profisetinidin, procyanidin and prodelphinidin. The average number of units varies from monomers to octamers with an average degree of polymerization(DPn) between 4 and 5 [15].

All of the other chemical material were purchased from Sigma-Aldrich (St. Louis, MO, USA). Sodium hydroxide (98%), epichlorohydrin and acetone were used as reagents in the epoxidation reaction. Acrylic acid (99%), hydroquinone (99%) and 1,4-diazabicyclo[2,2,2]octane (97%) were used for producing the bioepoxy resin.

### 2.2. Synthesis of Glycidyl Ether Tannin

Tannin was epoxidized with epichlorohydrin. Glycidyl ether tannin was obtained with epichlorohydrin at a reaction temperature of 80 °C and a ratio of tannin to NaOH (20% soluble in ethanol) of 1:1.5, respectively. Epichlorohydrin (15 g) was dissolved in water (10 g) at room temperature under mechanical stirring for 30 min. Subsequently, the mixture solution was placed in a 100-mL three-neck round-bottomed flask equipped with a reflux condenser with tannin (3 g) and heated to 80 °C under mechanical stirring. An aqueous sodium hydroxide solution of a 20 wt % concentration (4.5 g) was added dropwise using a dropping funnel while continuing to stir. The reaction was continued for 3 h to allow the reaction to occur. After reaction completion, the solution was diluted to 200 mL with acetone. The salts released as by-products in the reaction medium were filtered out over a 1-µL glass-fiber filter. The acetone, water and non-reacted excess of epichlorohydrin were evaporated using a rotary evaporator at 90 °C under reduced pressure. The reaction product was then redissolved in acetone (40 mL), filtered over a 1-µL glass-fiber filter, and the filtrate was evaporated under vacuum at 90 °C under reduced pressure. This last step was repeated twice. The remaining product was glycidyl ether tannin (GET). The appearance of GET was a light brown-colored liquid. Its viscosity was measured with a Brookfield RV viscometer (Harlow, UK), yielding a viscosity of 760 centipoises with spindle No. 5 and a 60 rpm rate; the pH was 10.3, and the epoxy index was 7.2% [6].

### 2.3. Synthesis Tannin Epoxy Acrylate

Bio tannin-based epoxy resin was synthesized from GET as the core of the molecule and acrylic acid, catalyst and hydroquinone. Hydroquinone inhibits the self-reaction of acrylic acid. The catalyst was used to accelerate the reaction. For producing the bio-tannin epoxy acrylate resin, acrylic acid (1.5 mL) was dissolved in hydroquinone (0.04 g) and 1,4-diazabicyclo[2,2,2]octane (0.06 g). After thoroughly mixing at ambient temperature, the mixture was placed in a 100-mL three-neck round-bottomed flask with a magnetic stirrer, thermometer and GET (4 g). The reaction mixture was heated under two variable reaction times and reaction temperatures. To determine the more suitable reaction parameters, the conditions used were a combination of two temperatures, namely 80 and 95 °C, and two reaction times, namely 8 and 12 h. The finished products were obtained as viscous dark-brown liquids. The variables of the synthesis of these bio-based tannin epoxy acrylate resins were the 4 resins prepared respectively at 80 °C for 12 h (resin ET1), at 80 °C for 8 h (ET2), at 95 °C for 12 h (ET3) and 95 °C for 8 h (ET4). The epoxy index (epoxide equivalent/kg of resin) was determined by chemical analysis using a method described in the literature [16]. When epoxies are present in the reaction media, the addition of HBr leads to opening of the epoxy groups. When all of the epoxy groups are opened by HBr, the further addition of HBR results in the blue-green end of the crystal violet indicator. The epoxy index is then calculated as:

Epoxy % = (*v* × *n* × 1.6)/*W*_m_ (1)

where *v* is the titration solution consumed, *n* is the normalized value and *W*_m_ is the amount of glycidyl ether tannin. For the four resins, the epoxy indexes were: resins at 80 °C for 12 h (ET1) (epoxy index = 6.4), at 80 °C for 8 h (ET2) (epoxy index = 7.2), at 95 °C for 12 h (ET3) (epoxy index = 4.8), and 95 °C for 8 h (ET4) (epoxy index = 5.6).

The resins synthesis was also followed by titration. The resin was weighed in an Erlenmeyer flask and dissolved in ethanol. Additionally, two drops of phenolphthalein were added. A 0.1 M KOH solution was used to titrate. The end point was taken when the solution turned from pink to yellow. The acid value was calculated as:

Acid Value = *V*_titre_ × KOH normality/mass of sample (2)

The acid value of the resins was 10–12.

### 2.4. FTIR Analysis

All of the bio tannin epoxy acrylate resins were analyzed with a Perkin Elmer Frontier Attenuated Total Reflectance-Fourier Transform-Mid InfraRed (ATR-FT-MIR) spectrometer (Waltham, MA, USA) provided by an ATR Miracle diamond crystal. The liquid samples were placed on the diamond eye (1.8 mm) of the ATR equipment, and the contact for the sample was ensured by tightly screwing the clamp device. Each resin was scanned registering the spectrum with 32 scans with a resolution of 4 cm^−1^ in the wave number range between 600 and 4000 cm^−1^. Each sample from the tannin-based epoxy resin was scanned five times, and the average of these spectra after vector normalization and baseline correction was studied in the fingerprint region between 1800 and 600 cm^−1^.

### 2.5. ^13^C-NMR Spectroscopy

^13^C-NMR DEPT spectra were recorded on a Bruker MSL 300 spectrometer (Bruker France, Wissembourg, France) at a frequency of 75.47 MHz. Chemical shifts were calculated relative to tetramethyl silane (TMS). The rotor was spun at 12 kHz on a double-bearing 4-mm Bruker probe (Bruker France, Wissembourg, France). The spectra were acquired with 5-s recycle delays, a 90° pulse of 5 µs and a contact time of 1 ms. The number of transients was 3000. The spectra were run with the suppression of spinning side bands.

### 2.6. MALDI-TOF Mass Spectrometry

The spectra were recorded on a KRATOS AXIMA Performance MALDI instrument (Kratos Analytical, Shimadzu, Manchester, UK). The irradiation source was a pulsed nitrogen laser with a wavelength of 337 nm. The length of one laser pulse was 3 ns. The measurements were carried out using the following conditions: polarity positive, flight path-linear, mass-high (20-kV acceleration voltage), 100–150 pulses per spectrum. The delayed extraction technique was used applying delay times of 200–800 ns. The samples were dissolved in acetone (4 mg/mL). The sample solutions were mixed with an acetone solution (10 mg/mL acetone) of the matrix. As the matrix, 2,5-dihydroxy benzoic acid (DHB) was used. For the enhancement of ion formation, NaCl was added to the matrix. The solutions of the sample and the matrix were mixed in equal amounts, and 0.5–1 μL of the resulting solution were placed on the MALDI target. After evaporation of the solvent, the MALDI target was introduced into the spectrometer. There is no interference noticeable of the DHB on the spectrum, because this should result at 154 + 23 Da (Na^+^), and the MALDI were done with the ion gate at 200 Da. This eliminates any lower molecular weight. The use of the ion gate is applied to be sure to identify the oligomers formed without interference from lower molecular weight species.

### 2.7. Preparation of the Adhesive Mixtures and Bonding of Wood Specimens

Prior to bonding, all of the beech wood blocks, of dimensions of 150 × 20 × 5 mm^3^, were planned in order to ensure freshly-cut, smooth and flat surfaces. The beech wood had been preconditioned at 8% moisture content for 6 weeks before the tests. The wood blocks were then bonded together two by two with the different adhesives. Each of the adhesives was applied by means of a roller, using an application rate of 200 g/m^2^. The blocks were pressed together each by means of two clamps to have self-equilibration of the amount of resin used and to have close contact glue lines of ≤0.1-mm thickness according to European Norm EN 12765 (2002) [17]. The tannin epoxy-acrylate-bonded samples were cured at 60 °C for 24 h in an oven.

### 2.8. Block Shear Strength Test

The shear strength of the specimens bonded with bio tannin-based epoxy acrylate resin was determined by the test defined in European Norm EN 12765:2002 [17]. The universal testing machine used was a constant-rate-of-traverse machine. It was an INSTRON-4467 (Instron, High Wycombe, UK). Five bonded samples for each resin type were cut into specimens for the shear test according to the standard. The dimensions of the shear areas were measured for all of the specimens, and the strength in Newtons per square millimeter (N/mm^2^) was calculated using Equation (3):

(3) $$\tau =\frac{F_{\max}}{A}=\frac{F_{\max}}{l_{2}\times b}$$

where

*F*_max_ = the applied maximum force in Newtons (N);
*A* = the bonded test surface in square millimeters (mm^2^);
*l*_2_ = the length of the bonded test surface in millimeters (mm);
*b* = the width of the bonded test surface in millimeters (mm).

## 3. Results and Discussion

### 3.1. Synthesis of the Tannin-Based Bioepoxy Acrylate Resin

As show in Figure 1, in the epoxidation reaction, the hydroxyl groups on the ring of the tannin reacted with epichlorohydrin to obtain glycidyl groups under alkaline reaction conditions.

**Figure 2:**
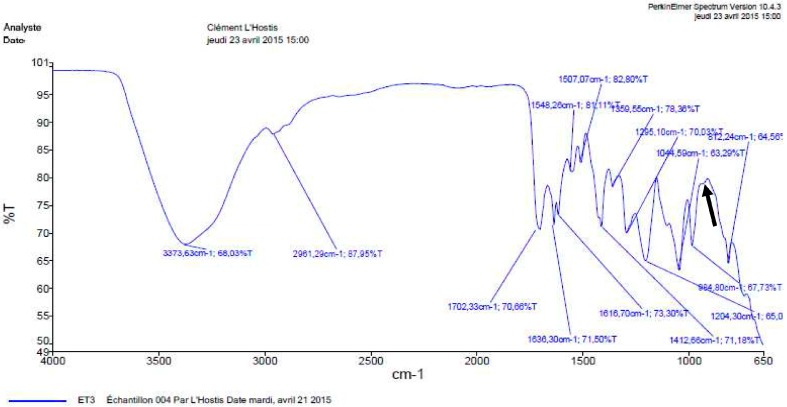
FTIR spectrum of the ET3 tannin epoxy acrylic resin sample.

Since epoxy groups are unstable, by opening the epoxy ring, and in the presence of a catalyst, reaction ensues with acrylic acid. The reaction scheme of glycidylation of tannin (GET) with acrylic acid is shown in Figure 2. The GET can react with acrylic acid through the following three reactions:

I. Additional esterification of epoxy and acid groups.

II. Condensation esterification of secondary hydroxyl groups in the epoxy chain with products of the reaction of the acid and epoxy and acid groups.

III. Etherification of epoxy groups and secondary hydroxyls in the epoxy chain with products of reaction acid and epoxy and acid groups.

For the synthesis of epoxy acrylates, only the first reaction is desired. Under controlled reaction conditions (temperature and reaction time) and condensing and returning the water vapor formed to the reaction system, it was expected that the unwanted reactions (Types II and III) did not occur during the resin synthesis. The additional esterification reaction of GET groups and acrylic acid groups led to the loss of epoxy groups and the formation the carbonyl ester bond (links to vinyl bonds) and secondary hydroxyl groups. The spectroscopy methods were used to monitor the decrease and elimination of epoxy groups and the formation hydroxyl group and double-bond ester functions after the formation of the epoxy tannin acrylate.

### 3.2. FT-MIR Analyses of Tannin Epoxy Acrylate Resin

Figure 2 shows the FT-MIR spectrum for the ET3 resin. Analysis of this FTIR spectra suggested that the product formed during the reaction. Thus, epoxide signals due to ring vibrations appeared at 800–900 and 1044–1295 cm^−1^. The substrate (tannin) showed typical absorption for aromatic and aliphatic hydroxyl groups stretching over 3300, 1400–2000 and another at 1359 cm^−1^ corresponding to aromatic OH deformation. The methyl group of methyloxirane moieties signals appeared at 2960 cm^−1^. The observed signals are detailed in Table 1. The overall comparison of the four reaction products indicates that the presence of methyloxirane groups confirms that substitution has occurred during the course of the reaction. Furthermore, the band intensity of the ET3 sample increased in relation to the other samples, confirming that increasing the temperature and time of reaction led to improved substitution of the starting material. The spectrum of the epoxy-acrylic resin sample in Figure 2 can be compared to the spectrum of the tannin-epoxy resin shown in [6]. The shifts characteristic of the oxirane ring on epoxidized flavonoids were found to be in a previous study at 905 and 835 cm^−1^ [6]. On the FTIR spectrum in Figure 2, these two bands have one disappeared and the other one (the 835 cm^−1^) almost disappeared (*cf.* the black arrow in Figure 2). Table 1 shows the interpretation of the peaks of the spectra for the ET3 tannin epoxy acrylate resin.

### 3.3. ^13^C-NMR Analysis

**Scheme 1:**
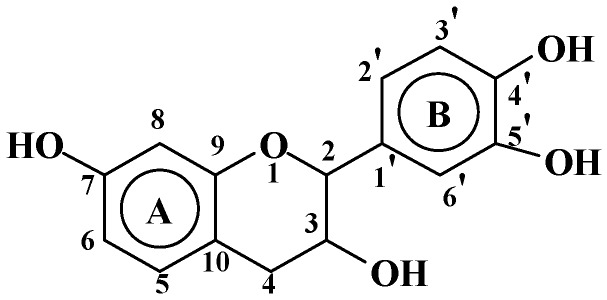
Sites numbering of a flavonoid unit.

**Figure 3:**
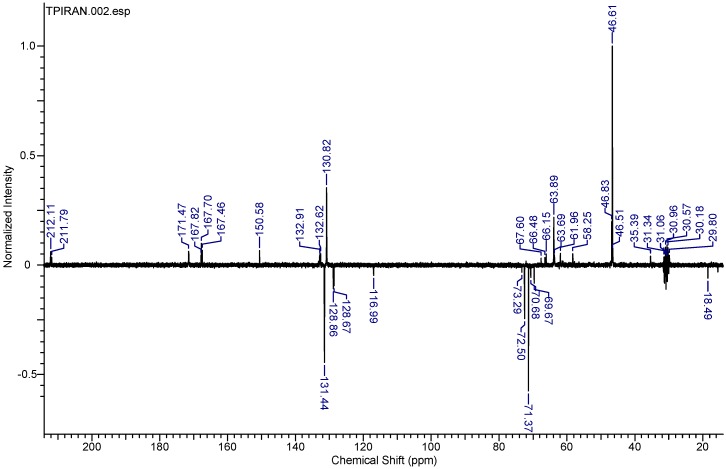
^13^C-NMR spectrum of the ET3 tannin epoxy acrylate resin.

The ^13^C NMR spectra were done as distortion enhancement by polarization transfer 135° (DEPT) spectra indicating that several groups have formed in the reaction of tannin with epichlorydrin under the conditions indicated. Scheme 1 indicates the sites numbering of a flavonoid unit. The NMR spectrum of ET3 tannin epoxy acrylic resin is shown in Figure 3. The most indicative peak showing that epoxy groups that have been formed and have reacted with the opening and further reaction is the absence of the peak at 46.4 ppm for the –CH_2_-epoxy group and the appearance of the 46.5-, 46.6- and 46.8-ppm peaks belonging to the –CH_3_ groups formed from it. None of the other confirming peaks determined in a previous investigation of GET [6] indicating the presence of an epoxy ring are present, further confirming its further reaction.

The 131.4-ppm peak belongs to the –CH_2_– groups of either a –O–CH_2_–CHOH–CH_3_ group to a –O–CH_2_–CHOH–CH_2_– bridge. That both of these exist is indicated by the fairly small –CH_3_ peak at 31 ppm, the probability of the –O–CH_2_–CHOH–CH_2_– bridge linking two flavonoids or flavonoid oligomers having already been proven in a previous investigation on GET [6], however, to be the dominant one. This indicates a phenolic site of the flavonoid where an epoxy group got attached and that has further reacted by opening the epoxide and even further reacting with another flavonoid to bridge and link the two.

The peak at 70.7 ppm is the shift of the –CH_2_– group of the same –O–CH_2_–CHOH–CH_3_ or –O–CH_2_–CHOH–CH_2_– with the peaks at 66–67 ppm being the signals of the CH groups of the same.

The peak at 130.8 belongs to the CH group of acrylic acid attached to the C=O group and the peak at 131.4 ppm to the CH_2_= group of acrylic acid. However, its shift at 131.4 ppm rather than the expected 133 ppm indicates that the acrylic acid double bond has opened as a consequence of a reaction. The C=O of unreacted acrylic acid is found at 171.4 ppm. The peaks at 71–73 ppm particularly evident in resin ET3 belong respectively to the –CH_2_– groups linked to a –O– of both of the original CH_2_= group of the acrylic acid after opening of the double bond and of the –CH_2_– group attached to the –O– of the opened epoxy ring, both being the consequence of the reaction of the acrylic acid with the epoxy ring.

All of the above indicates that a number of different species obtained by the reaction of the epoxy ring of GET with the acrylic acid does occur and coexist.

**Scheme 2:**
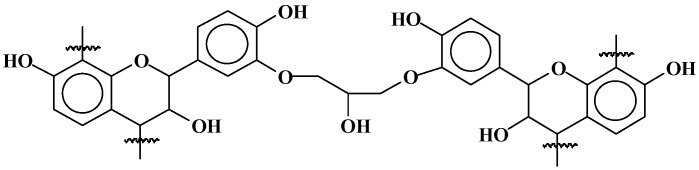
Epoxy tannin oligomers as previously identified.

**Figure 4:**
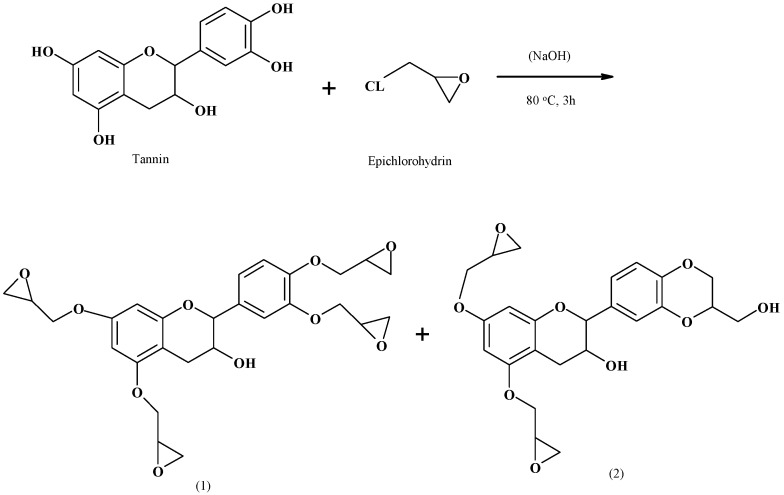
Reaction scheme for the epoxidation of tannin. Formation of epoxy groups on different sites of tannin’s flavonoid units.

Oligomers, such as already identified in previous work [6], also still subsist in the reacted mixture, such as the one shown in Scheme 2:

All of these coexist with unreacted flavonoid oligomers. Thus, the peaks at respectively 167.4–167.8 ppm (C7), 150.6 ppm (C3′/C4′), 132 ppm (C1′), 116.99 ppm (C2′), 108.6 ppm and 102 ppm are characteristic of flavonoids being, respectively, the C3′/C4′, the C1′ and the C2′. Figure 4 shows the ^13^C-NMR spectrum of the ET3 sample of tannin epoxy acrylate resin.

### 3.4. MALDI-TOF Mass Spectrometry

**Figure 5:**
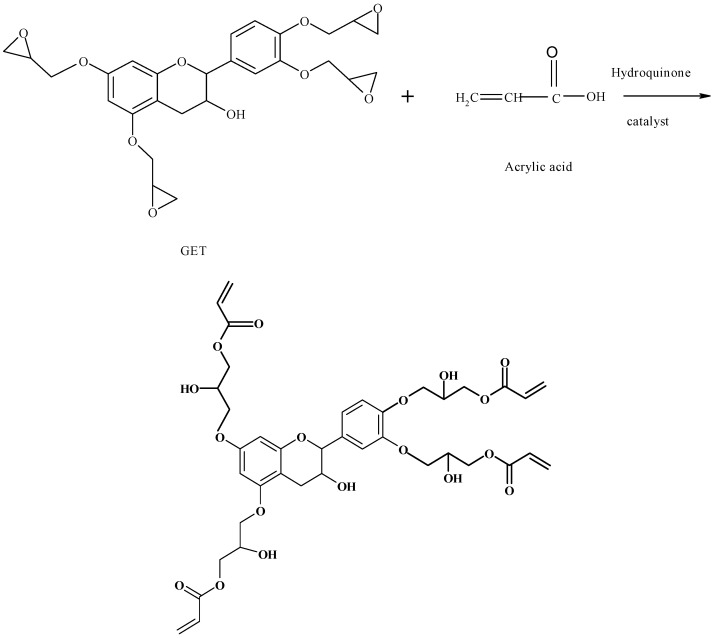
Reaction scheme of glycidylation of the tannin with acrylic acid (tannin-based epoxy acrylate).

MALDI-TOF mass spectrometry was used to ascertain if the expected reactions between GET and acrylic acid did occur and if the expected oligomers were formed. This analysis confirmed the ^13^C NMR analysis by indicating that tannin-based epoxy acrylates were prepared. The type of products expected and the reactions involved are shown in Figure 4 and Figure 5.

Reaction 1: 

Reaction 2:

The products of Reaction 1 in Figure 4 and Figure 5 and similar oligomers have already been observed in previous work [6], while it was hoped to prepare products similar to what is shown in Reaction 2.

**Scheme 3:**
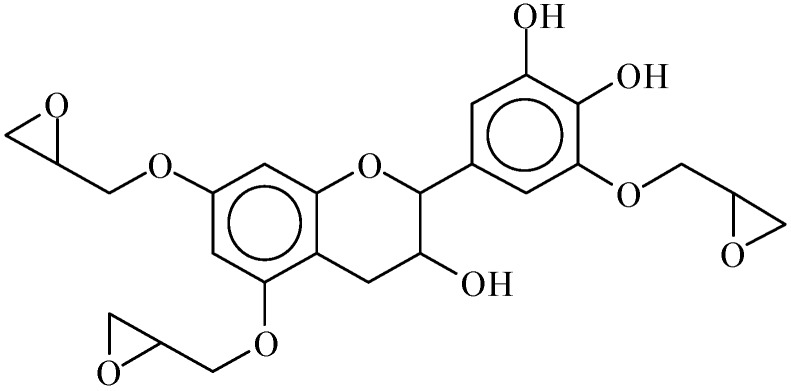
Most probable structure of the 476 Da MALDI peak.

**Scheme 4:**
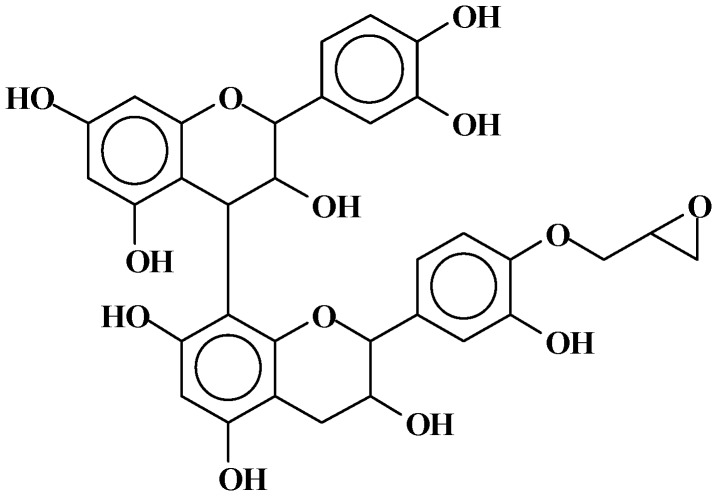
Catechin dimer with one epoxy group representing the 654 Da MALDI peak.

**Scheme 5:**
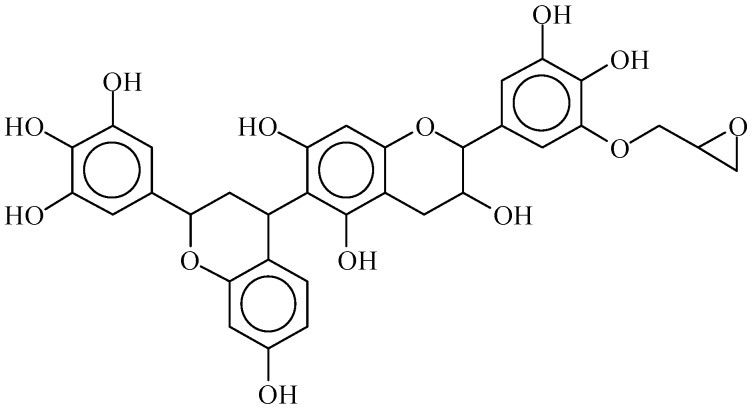
Robinetinidin-gallocatechin dimer with one epoxy group equally representing the 654 Da MALDI peak.

**Figure 6:**
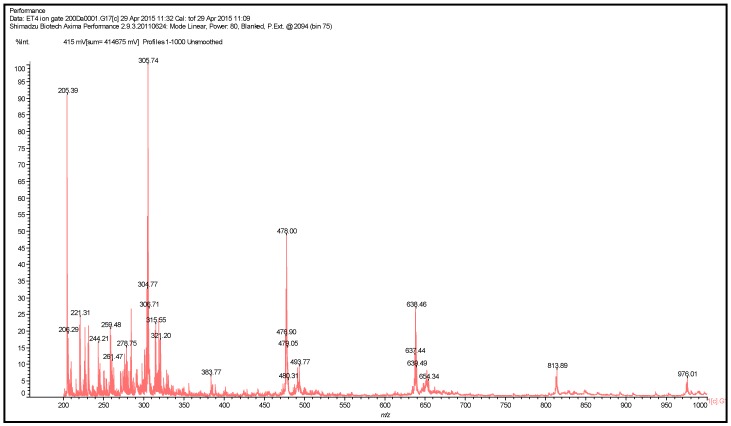
MALDI-TOF spectrum of the ET3 sample of tannin epoxy acrylate resin.

In the MALDI spectrum in Figure 6, a number of monomer and oligomer species of a different nature can be observed, these being listed in Table 2; thus, (i) flavonoid monomers, such as fisetinidin, catechin/robinetinidin and gallocatechin, and unreacted flavonoid dimers, such as the species at 638 Da; (ii) epoxidized flavonoids reacted with acrylic acid; and (iii) flavonoid monomers epoxidized, but not yet reacted with acrylic acid, such as the species represented by the peaks at 384, 476 Da, the most probable structure of which is (Scheme 3):

and 654 Da (Table 2), this latter one being a protonated catechin or robinetinidin dimer with one epoxy group, such as in Scheme 4: 

or a robinetinidin-gallocatechin dimer with one epoxy group, such as in Scheme 5:

The difference between the two being just the C4–C8 or C4–C6 interflavanoid linkage, or even a mixed catechin-robinetinidin dimer with one epoxy group.

**Scheme 6:**
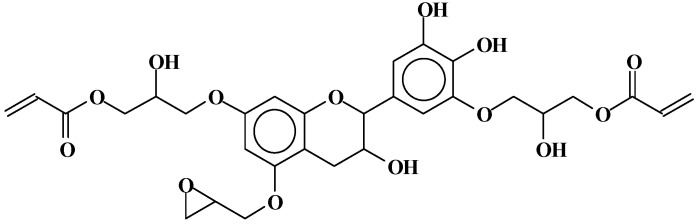
Chemical structure describing the 638-639 Da MALDI peak.

The MALDI spectrum does not allow one to determine on which site the epoxide is placed, as all different hydroxyl groups of the flavonoid are liable to have reacted. However, species where pre-epoxidized flavonoid monomers or dimers have reacted with acrylic acid do occur. In these species, either all of the epoxy groups have reacted with acrylic acid or, more commonly, part of the epoxy groups are still extant, while some others have indeed reacted with acrylic acid. One example of these mixed species is the one represented by the peak at 638–639 Da, where a gallocatechin three-epoxidized one has two epoxy groups having reacted with acrylic acid, while one is still an unreacted epoxy group (+Na^+^) and one or two hydroxyl groups are deprotonated (Scheme 6).

Again, the MALDI spectrum does not allow one to determine on which of the hydroxyl group sites of the flavonoid the different reagents have reacted.

**Scheme 7:**
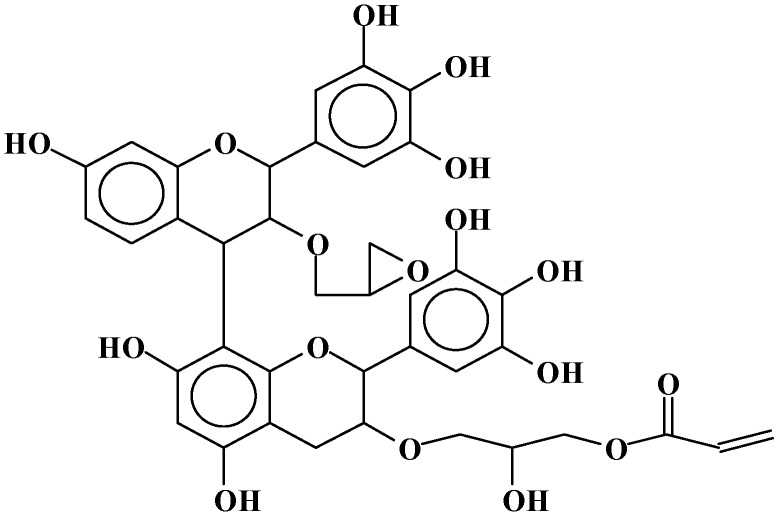
Chemical structure describing the 813 Da MALDI peak.

**Scheme 8:**
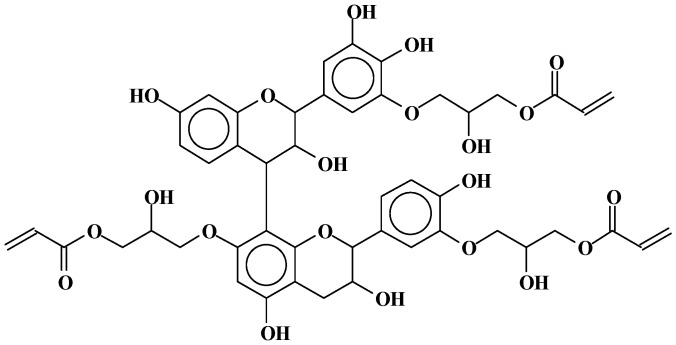
Chemical structure describing the 976 Da MALDI peak.

Equally, the species corresponding to the 813-Da peak, a dimer of either robinetinidin-gallocatechin or catechin-gallocatechin, also falls into this category (Scheme 7):

Species in which all epoxy groups have reacted with acrylic acid also occur. This is the case of the robinetinidin-catechin, or catechin-catechin, or robinetinidin-robinetinidin dimer at 976 Da (without Na^+^), one of the possible structures of which can be represented as (Scheme 8):

where all three epoxy groups have reacted with acrylic acid.

However, the structure represented by the 976-Da peak can also just be a gallocatechin-catechin-gallocatechin trimer with one epoxy group (+Na^+^). Any one of these two structures is possible.

MALDI-TOF mass spectrometry confirms that the reaction between GET and acrylic acid has occurred and that combined tannin epoxy acrylate resins have formed as observed by ^13^C-NMR. The MALDI-TOF spectrum of the ET3 tannin epoxy acrylate resin is shown in Figure 6. The oligomers formed are shown in Table 2.

### 3.5. Tensile Shear Test

The shear strength of the specimens bonded with different bio-based tannin epoxy acrylate adhesive resins is shown in Table 3. As can be seen, shear strength values of the different adhesive types were 7.5, 6.4, 10 and 8.9 (N/mm^2^), respectively. The shear strength values of sample ET3 were better than those of the other samples. This indicates that epoxy acrylate adhesive resins based on commercial tannin extracts, thus not on pure tannins, but still on a natural material, are conceivable as wood adhesives. Increases in reaction time and reaction temperature to prepare bio-based tannin epoxy acrylate resins have a positive effect on the bonding strength of the final resin.

## 4. Conclusions

Matrix-assisted laser desorption/ionization time-of-flight (MALDI-TOF) mass spectrometry coupled with ^13^C NMR was a suitable method for the analysis of the reaction products formed by the reaction of bioepoxy tannins with acrylic acid. They have been shown to be capable of determining the oligomers formed and the oligomers’ distribution in relation to their epoxy structure and characteristics. The results of this study according to MALDI-TOF mass spectrometry, ^13^C-NMR and FT-MIR spectroscopy showed that the best condition for prepare synthesis bioepoxy resin without bisphenol A with GET and acrylic acid was a reaction temperature of 95 °C for 12 h. The results of wood block shear strength tests of tannin epoxy acrylate adhesive resins indicated that these were the better conditions under which to prepare these resins.

## Figures and Tables

**Table 1:** Assignment of groups in the FTIR spectrum of the ET3 tannin epoxy acrylate resin.

Band (cm^−1^)	Assignment
3,300	–OH stretching
2,960	C–H stretching of –CH_2_ in methyloxirane (epoxy)
1,702	CO stretching in acrylic acid
1,616	C=C stretching in aromatic ring
1,507	C–C stretching of aromatic ring
1,359	Aromatic –OH
1,295	Stretching of cyclic ether (epoxy)
1,044	C–O–C ethers stretching
905	Oxirane (epoxy) group C–O stretching
835	C–O–C in oxirane group

**Table 2:** Monomer and oligomer species observed from the MALDI spectrum of the ET3 epoxy tannin acrylic resin.

Peak	Chemical species
278 Da	Fisetinidin monomer without Na^+^
305.7 Da	Gallocatechin monomer without Na^+^
315.6 Da	Catechin or robinetinidin monomer with Na^+^
328 Da	Gallocatechin monomer with Na^+^
384 Da	Gallocatechin monomer reacted with one epoxy group with Na^+^
476 Da	Three epoxy groups-gallocatechin monomer, protonated without Na^+^
478 Da	Three epoxy groups-catechin monomer, deprotonated with Na^+^
638 Da	Two acrylates-one epoxy gallocatechin monomer, deprotonated with Na+
654.3 Da	One epoxy gallocatechin-catechin dimer, deprotonated with Na+
813 Da	one epoxy-one acrylate-robinetinidin-gallocatechin dimer with Na^+^
976 Da	catechin-robinetinidin dimer co-reacted with 3 acrylics without Na^+^

**Table 3:** Shear strength of block shear wood joints tested according to norm EN 12675:2002 [13].

Resin type	Shear strength (N/mm^2^)	Standard deviation	Wood failure (%)
ET1	7.5	0.6	68
ET2	6.4	0.4	55
ET3	10.0	1.1	100
ET4	8.9	0.8	92
